# Translating patient-reported outcome measures: a multi-step process is essential

**DOI:** 10.1590/S1806-37132014000300002

**Published:** 2014

**Authors:** Catherine Acquadro, Ana Bayles, Elizabeth Juniper

**Affiliations:** Mapi, Lyon, France; Mapi, Lyon, France; Department of Clinical Epidemiology and Biostatistics, McMaster University, Canada

What does "translating" mean? Whilst theories are discussed elsewhere,^(^ ^1^ ^)^ the definition given by Umberto Eco^(^ ^2^ ^)^ seems refreshing and sensible: translating means "saying almost the same thing." What, however, is the extent of "almost" and how do you evaluate it? According to Eco, being faithful to a source document is not performing a "word for word" translation but a "world for world" translation and negotiating with the requirements of the source world becomes the key issue. In other words, the elasticity of almost depends on criteria that should be discussed and defined before embarking on the translation as such and in collaboration with the author of the original text.

When preparing patient-reported outcome (PRO) instruments for use internationally, it is helpful to remember Umberto Eco's observation. Regulators have focused their interest on the validity of the translations and their ability to express and investigate equivalent concepts across all language versions. With the question: "Are health-related quality of life (HRQoL) instruments internationally validated?,"^(^ ^3^ ^)^ the European Medicines Agency clearly made the aspect of equivalence one of the key issues of HRQoL evaluation. The US Food and Drug Administration shares this view in its guidance, section III.G.3., and provides some recommendations.^(^ ^4^ ^)^ The guidance states: "Regardless of whether the instrument was developed concurrently in multiple cultures or languages or whether a fully developed instrument was adapted or translated to new cultures or languages, we recommend that sponsors provide evidence that the content validity and other measurement properties are adequately similar between all versions used in the clinical trial. We will review the process used to translate and culturally adapt the instrument for populations that will use them in the trial."

Several reviews^(^ ^5^ ^,^ ^6^ ^)^ suggest that using a rigorous and multi-step process with centralized review procedures may lead to better translations of PRO measures and can meet the regulators' requirements stated above. Usually, the process of translating PRO measures involves the following steps: translation of the original instrument into the target language by two independent translators and reconciliation into one version (forward step); translation of the reconciled version back into the language of the original instrument (backward step); review of the reconciled version with the participation of the developer of the original instrument; test on a panel of patients living in the target country (cognitive interview step); review of the test by a panel of experts; and finalization of the translated version of the instrument. Of course, all of those steps should always be preceded by one crucial step: one should always request permission to translate from the developer of the original instrument, to prevent any misuse or modifications that would impair the right of the original developer to the integrity of the instrument.^(^ ^7^ ^)^

As previously mentioned, equivalence in content validity between the original and the translated versions is crucial. Validating the content of a PRO measure requires providing evidence that the questionnaire contains all the problems that are most important to the patients who are going to complete the questionnaire. Cultural and environmental (i.e., climate) issues have to be taken into consideration (e.g., going to market/doing one's shopping may be a problem in many countries but not so much in the United States, where most people drive; in tropical countries, asthma patients do not have to contend with snow and icy winds). Therefore the cognitive interviews with the patients must cover not only issues of comprehension but also cultural and environmental issues. Relevance of the questions to respondents should be checked and those that are obviously lacking from the perspective of content validity should be changed. For example, in the Pediatric Asthma Caregiver's Quality of Life Questionnaire (PACQLQ), which was developed in Canada, caregivers reported that they were "angry" because their child had asthma. Therefore, this concept was included in the original PACQLQ. However, in every other country in the world, that is not an emotion that is frequently experienced by caregivers-instead, they are "sad". Translated versions of the PACQLQ include this concept of sadness.

In their paper entitled "Leicester Cough Questionnaire: translation to Portuguese and cross-cultural adaptation for use in Brazil", Felisbino et al.^(^ ^8^ ^)^ describe such a multi-step process and provide evidence that the Brazilian version of the Leicester Cough Questionnaire (LCQ) measures the same concepts as the original English version and can be widely used to assess the quality of life of patients with chronic cough in Brazil. They describe how the Brazilian version of the LCQ was created in collaboration with the developer of the original questionnaire to ensure that the intent of the original items was appropriately captured in the translation. They report that there were no difficulties in translating words referring to symptoms, physical activities, or activities of daily living. However, some English-language idioms and phrases, such as "fed up" and "overall enjoyment", were the objects of review and discussion. In addition, there was a need to adjust the verb tense so that the addressed situation made sense in Portuguese. Felisbino et al. also show that testing the translation on a panel of Brazilian patients was a crucial step in developing the final translated version of the LCQ. The questionnaire was administered to ten participants with chronic cough in order to determine its acceptability, clarity, and understandability. Although the participants had varied educational levels, no significant difficulties that would prevent them from understanding the questionnaire were identified. This indicates that the measure produced can be administered to individuals from various socioeconomic classes and cultural backgrounds. The analysis of the responses given during the cognitive debriefing process showed that few items needed to be revised because of problems related to understandability. This finding is of great relevance because it shows the robustness of the process of translation and cross-cultural adaptation. The next steps will involve clinical studies in patients with chronic cough to evaluate the psychometric properties (i.e., validity, reliability and responsiveness) of the Brazilian LCQ, with the objective of achieving properties similar to those of the original.
