# Supplementary material for: Translating patient-reported outcome measures: a multi-step process is essential
Source: J Bras Pneumol. 2014 May-Jun;40(3):211–2. doi: 10.1590/S1806-37132014000300002 (PMC4109191; doi:10.1590/S1806-37132014000300002)
Supplement: Translation

# Translation: Tradução de medidas de resultados relatados pelo paciente: um processo composto de várias etapas é essencial

**Authors:** Catherine Acquadro, Ana Bayles, Elizabeth Juniper

**Affiliations:** Mapi, Lyon, França; Mapi, Lyon, França; Departamento de Epidemiologia Clínica e Bioestatística, Universidade McMaster, Canadá

O que significa "traduzir"? Enquanto teorias são discutidas em outra publicação,^(^ ^1^ ^)^ a definição dada por Umberto Eco^(^ ^2^ ^)^ parece refrescante e sensata: traduzir significa "dizer quase a mesma coisa". Qual, porém, é a extensão do "quase" e como se avalia isso? De acordo com Eco, ser fiel a um documento de origem não é realizar uma tradução "palavra por palavra", mas sim uma tradução "mundo para mundo" e negociar com as exigências do mundo de origem torna-se uma questão-chave. Em outras palavras, a elasticidade do quase depende de critérios que devem ser discutidos e definidos antes de embarcar na tradução como tal e em colaboração com o autor do texto original.

Ao preparar instrumentos de resultados relatados pelo paciente (RRP) para uso internacional, é útil lembrar a observação de Umberto Eco. Órgãos reguladores têm centrado seu interesse na validade das traduções e sua capacidade de expressar e investigar conceitos equivalentes em todas as versões linguísticas. Com a pergunta: "Os instrumentos de qualidade de vida relacionada à saúde (QVRS) são validados internacionalmente?"^(^ ^3^ ^)^ a European Medicines Agency claramente tornou o aspecto da equivalência uma das questões-chave da avaliação da QVRS. A US Food and Drug Administration compartilha dessa visão em sua orientação, seção III.G.3., e fornece algumas recomendações.^(^ ^4^ ^)^ A orientação afirma: "Independentemente de o instrumento ter sido desenvolvido simultaneamente em várias culturas ou línguas ou de um instrumento totalmente desenvolvido ter sido adaptado ou traduzido para novas culturas ou línguas, recomendamos que os apoiadores forneçam evidências de que a validade de conteúdo e outras propriedades de medida são adequadamente semelhantes entre todas as versões utilizadas no ensaio clínico. Examinaremos o processo utilizado para a tradução e adaptação cultural do instrumento para populações que irão utilizá-lo no ensaio."

Diversas revisões^(^ ^5^ ^,^ ^6^ ^)^ sugerem que a utilização de um processo rigoroso e de várias etapas, com procedimentos centralizados de revisão, pode levar a melhores traduções de medidas de RRP e pode atender às exigências dos órgãos reguladores acima enunciadas. Geralmente, o processo de tradução de medidas de RRP envolve as seguintes etapas: tradução do instrumento original para a língua-alvo por dois tradutores independentes e reconciliação em uma versão (etapa progressiva); tradução da versão reconciliada de volta para a língua do instrumento original (etapa regressiva); revisão da versão reconciliada com a participação do idealizador do instrumento original; teste em um grupo de pacientes que vivem no país onde o instrumento será utilizado (etapa da entrevista cognitiva); revisão do teste por um grupo de especialistas; e finalização da versão traduzida do instrumento. É claro que todas essas etapas devem ser sempre precedidas por uma etapa crucial: deve-se sempre solicitar a permissão do idealizador do instrumento original para traduzir o mesmo, a fim de evitar qualquer uso indevido ou modificações que possam prejudicar o direito do idealizador original à integridade do instrumento.^(^ ^7^ ^)^

Como mencionado anteriormente, a equivalência na validade de conteúdo entre as versões original e traduzida é crucial. A validação do conteúdo de uma medida de RRP exige o fornecimento de evidências de que o questionário contém todos os problemas que são mais importantes para os pacientes que vão preencher o questionário. Questões culturais e ambientais (isto é, o clima) têm que ser levadas em consideração (por ex., ir ao mercado/fazer compras pode ser um problema em muitos países, mas não tanto nos Estados Unidos, onde a maioria das pessoas dirige; em países tropicais, os asmáticos não têm que lidar com neve e ventos gelados). Portanto, as entrevistas cognitivas com os pacientes devem cobrir não apenas questões de compreensão, mas também questões culturais e ambientais. Deve-se checar a relevância das perguntas para os entrevistados, e aquelas que forem obviamente deficientes sob a perspectiva da validade de conteúdo devem ser modificadas. Por exemplo, no Pediatric Asthma Caregiver's Quality of Life Questionnaire (PACQLQ, Questionário de Qualidade de Vida de Cuidadores de Crianças com Asma), que foi desenvolvido no Canadá, os cuidadores relataram ficar "zangados" pelo fato de a criança ter asma. Portanto, esse conceito foi incluído no PACQLQ original. Porém, em vários países do mundo, essa não é uma emoção comumente experimentada pelos cuidadores - em vez disso, eles ficam "tristes". Versões traduzidas do PACQLQ incluem esse conceito de tristeza.

Em seu trabalho intitulado "Leicester Cough Questionnaire: translation to Portuguese and cross-cultural adaptation for use in Brazil" (Questionário de Leicester sobre tosse crônica: tradução e adaptação cultural para a língua portuguesa falada no Brasil), Felisbino et al.^(^ ^8^ ^)^ descrevem um processo assim composto de várias etapas e fornecem evidências de que a versão brasileira do Leicester Cough Questionnaire (LCQ) mede os mesmos conceitos que a versão original em inglês e pode ser amplamente utilizada para avaliar a qualidade de vida de pacientes com tosse crônica no Brasil. Descrevem como a versão brasileira do LCQ foi criada em colaboração com o idealizador do questionário original para garantir que a intenção dos itens originais foi devidamente captada na tradução. Relatam que não houve dificuldades na tradução de palavras referentes a sintomas, atividades físicas ou atividades da vida diária. Porém, algumas expressões idiomáticas e frases da língua inglesa foram motivo de revisão e discussão, entre elas, "fed up" e "overall enjoyment". Além disso, houve necessidade de adequação do tempo verbal para que a ação questionada fizesse sentido na língua portuguesa. Felisbino et al. também mostram que testar a tradução em um grupo de pacientes brasileiros foi uma etapa crucial na elaboração da versão final da tradução do LCQ. O questionário foi aplicado a dez participantes com tosse crônica para se determinar sua aceitabilidade, clareza e compreensibilidade. Embora os participantes apresentassem níveis de escolaridade variados, não foram detectadas dificuldades significativas que impedissem a compreensão do mesmo. Isso indica que a medida produzida pode ser aplicada a indivíduos de diferentes classes socioeconômicas e origens culturais. A análise das respostas dadas durante o processo de desdobramento cognitivo demonstrou a necessidade de reavaliação de poucos itens em razão de problemas relacionados à compreensibilidade. Esse achado é de grande relevância, uma vez que mostra a robustez do processo de tradução e de adaptação cultural. As próximas etapas envolverão estudos clínicos em pacientes com tosse crônica para avaliar as propriedades psicométricas (isto é, a validade, confiabilidade e responsividade) do LCQ brasileiro, com o objetivo de atingir propriedades semelhantes às do original.
